# Kushenol A and 8-prenylkaempferol, tyrosinase inhibitors, derived from *Sophora flavescens*

**DOI:** 10.1080/14756366.2018.1477776

**Published:** 2018-06-06

**Authors:** Jang Hoon Kim, In Sook Cho, Yang Kang So, Hyeong-Hwan Kim, Young Ho Kim

**Affiliations:** a Advanced Radiation Technology Institute, Korea Atomic Energy Research Institute, Jeongeup, Republic of Korea;; b Department of Horticultural and Crop Environment, National Institute of Horticultural and Herbal Science, RDA, Wanju, Republic of Korea;; c College of Pharmacy, Chungnam National University, Daejeon, Republic of Korea

**Keywords:** *Sophora flavescens*, Fabaceae, tyrosinase inhibitor, molecular docking, antioxidant

## Abstract

Tyrosinase is known for an enzyme that plays a key role in producing the initial precursor of melanin biosynthesis. Inhibition of the catalytic reaction of this enzyme led to some advantage such as skin-whitening and anti-insect agents. To find a natural compound with inhibitory activity towards tyrosinase, the five flavonoids of kushenol A (**1**), 8-prenylkaempferol (**2**), kushenol C (**3**), formononetin (**4**) and 8-prenylnaringenin (**5**) were isolated by column chromatography from a 95% methanol extract of *Sophora flavescens*. The ability of these flavonoids to block the conversion of L-tyrosine to L-DOPA by tyrosinase was tested *in vitro*. Compounds **1** and **2** exhibited potent inhibitory activity, with IC50 values less than 10** **µM. Furthermore, enzyme kinetics and molecular docking analysis revealed the formation of a binary encounter complex between compounds **1–4** and the enzyme. Also, all of the isolated compounds (**1–5**) were confirmed to possess antioxidant activity.

## Introduction

The root of *Sophora flavescens*, also known as Kushen, is one of approximately 50 species belonging to the Sophora genus of the Fabaceae family^1^. It is widely distributed in Asia, Oceanica and the Pacific Islands^1^, and has been used as a traditional herbal medicine for the treatment of viral hepatitis, fever, dysentery, cancer, myocarditis, scabies and pain^1–3^. Alkaloids and flavonoids are the main components of *S. flavescens*^4^. The quinolizidine alkaloids sophoridine, matrine, and oxymatrine exert anti-hepatitis B viral effects^4^. The prenylated flavonoids isoxanthohumol, norkurarinone, kurarinone, kushenol T, kuraridin, and norkurarinol exhibit anti-inflammatory, α-glucosidase inhibitory, free radical scavenging and vasorelaxant properties^1^^,^^5^^,^^6^.

Tyrosinase (EC 1.14.18.1) is a multifunctional copper-containing enzyme that is important for melanin biosynthesis related to pigment production in the skin, the browning of vegetables and cuticle formation in insects^7^^,^^8^. Two coppers of this enzyme participate in the conversion of L-tyrosine to 3,4-dihydroxy-L-phenylalanine (L-DOPA) during the early stage of melanin biosynthesis, as well as the conversion of L-DOPA to dopaquinone, the precursor of melanin^8^^,^^9^. At present, tyrosinase is a target for the development of skin-whitening agents in cosmetics and for insect control^8^^,^^9^. Natural science researchers have put forth continued efforts to develop tyrosinase inhibitors to solve these problems. Therefore, kojic acid, derived from Aspergillus oryzae or fermented rice, was developed as a representative inhibitor^10^. β-Arbutin was also found to be a potential inhibitor that can be isolated from natural plants^11^.

*Sophora flavescens* was examined previously for the identification of potential inhibitors of tyrosinase, which included sophoraflavanone G, kuraridin, kuraridinol, trifolirhizin and kurarinol, with IC50 values in the micromolar range^12^^,^^13^. The present study aimed to isolate additional prenylated and lavandulyl flavonoids to determine their inhibitory effects on the catalytic action of tyrosinase, utilising molecular docking analysis, and to evaluate their antioxidant activities.

## Materials and methods

### General experimental procedures

NMR experiments were conducted on an ECA500 (JEOL, Japan) spectrometer, with the chemical shift referenced to the residual solvent signals, using methanol-d_4_ as solvent. Mass spectra were measured using a Prominence TM UFLC system (Shimadzu, Kyoto, Japan). TLC analysis was performed on silica gel 60 F254 and RP-18 F254S plates (both 0.25** **mm layer thickness, Merck, Darmstadt, Germany); pure compounds were visualised by dipping plates into 10% (v/v) H_2_SO_4_ reagent (Aldrich, St. Louis, MO) and then heat treated at 110** **°C for 1** **min. Silica gel (Merck 60A, 70–230 or 230–400 mesh ASTM) and reversed-phase silica gel (YMC Co., ODS-A 12** **nm S-150, S-75** **μm) were used for column chromatography. 2,2′-Azino-bis(3-etheylbenzothiazoline-6-sulfonic acid (ABTS, A1888), tyrosinase (T3824) and L-tyrosine (T3754) were purchased from Sigma-Aldrich.

### Plant material

*Sophora flavescens* roots were purchased from herbal medicine market in Jeongeup (Korea, April 2015) and identified by one of authors (J.H. Kim). A voucher specimen (NIHHS-1) was deposited at the Herbarium, Department of Horticultural and Crop Environment, National Institute of Horticultural and Herbal Science.

### Extraction and isolation

*S. flavescens* roots (5** **kg) were extracted with 95% methanol (36** **L** **×** **2) at room temperature for a week. The methanol extract (770** **g) condensed under reduced pressure was suspended in distilled water (1** **L) and then progressively partitioned with chloroform (27** **g), ethyl acetate (100** **g) and water (600** **g) fractions. The ethyl acetate was subjected to a silica gel column chromatography with gradient system of chloroform/methanol (20:1 → 5:1) to obtain 10 fractions (E0.1–E0.10). E0.3 (7.0** **g) was separated using C-18 column chromatography with gradient system of methanol/distilled water (1:1 → 7:1) to give compound **1** (15.0** **mg) and five fractions (E.3.1–E.3.5). Compound **4** (8.0** **mg) and two fractions (E3.3.1–E3.3.2) were purified from E.3.3 (1.4** **g) on silica gel column chromatography with isocratic system of chloroform/methanol (35:65). E3.3.2 (0.3** **g) was subjected to C-18 column chromatography with gradient system of methanol/distilled water (1:1 → 7:1) to gain compound **5** (24.0** **mg). E0.7 (4.2** **g) was loaded on C-18 column chromatography and eluted with gradient system of methanol/distilled water (1:1 → 6:1) to obtain compound **3** (18.0** **mg) and four fractions (E.7.1–E.7.4). E.7.2 (0.7** **g) was chromatographied using a C-18 column chromatography and eluted with isocratic system of 65% methanol to gain compound **2** (20.0** **mg).

### Compound 1

White powder; ESI-MS *m/z*** **=** **407.2 [M-H]^–^, (calcd C_25_H_27_O_5_^–^, 407). ^1^H-NMR (500** **MHz, CD_3_OD) *δ* 7.54 (1H, dd, *J*** **=** **7.8, 1.4** **Hz, H-6′), 7.17 (1H, ddd, *J*** **=** **7.8, 7.8, 1.4** **Hz, H-4′), 6.90 (1H, ddd, *J*** **=** **8.2, 7.8, 0.98** **Hz, H-5′), 6.83 (1H, dd, *J*** **=** **8.2, 0.98** **Hz, H-3′), 5.90 (1H, s, H-6), 5.64 (1H, dd, *J*** **=** **11.7, 3.9** **Hz, H-2), 4.97(1H, overlapped, H-7″), 4.59 (1H, s, H-4a″), 4.53 (1H, s, H-4b″), 2.86 (2H, m, H-3), 2.60 (2H, m, H-1″), 2.48 (1H, m, H-2″), 2.01 (1H, m, H-6″), 1.63 (3H, s, H-5″), 1.55 (3H, s, H-9″), 1.46 (3H, s, H-10″). ^13^C NMR (125** **MHz, CD_3_OD) *δ* 198.6 (C-4), 166.8 (C-7), 163.3 (C-8a), 162.4 (C-5), 155.3 (C-2′), 149.8 (C-3″), 132.2 (C-8″), 130.2 (C-4′), 127.5 (C-6′), 127.2 (C-1′), 124.8 (C-7″), 120.7 (C-5′), 116.2 (C-3′), 111.3 (C-4″), 108.8 (C-8), 103.4 (C-4a), 96.6 (C-6), 75.9 (C-2), 43.1 (C-3), 32.3 (C-6″), 28.1 (C-2″), 25.9 (C-9″), 19.3 (C-5″), 17.9 (C-10″).

### Compound 2

Yellow powder; m.p. 147–149** **°C, ESI-Ms *m/z*** **=** **355.9 [M** **+** **H]^+^ (calcd C_20_H_19_O_6_^+^, 355). ^1^H-NMR (500** **MHz, CD_3_OD) *δ* 8.09 (2H, d, *J*** **=** **8.2** **Hz, H-2′,6′), 6.89 (2H, d, *J*** **=** **8.2** **Hz, H-3′,5′), 6.22 (1H, s, H-6), 5.21 (1H, t, *J*** **=** **6.3** **Hz, H-2″), 3.50 (2H, d, *J*** **=** **6.3** **Hz, H-1″), 1.80 (3H, s, H-5″), 1.67 (3H, s, H-4″). ^13^C NMR (125** **MHz, CD_3_OD) *δ* 177.8 (C-4), 163.1 (C-7), 160.7 (C-5), 160.2 (C-4′), 155.6 (C-8a), 148.1 (C-2), 137.0 (C-3), 132.5 (C-3″), 130.8 (C-2′,6′), 124.1 (C-2″), 116.4 (C-3′,5′), 107.8 (C-4a), 104.6 (C-8), 98.9 (C-6), 26.0 (C-4″), 22.6 (C-1″), 18.3 (C-5″).

### Compound 3

^1^H-NMR (500** **MHz, CD_3_OD) *δ* 7.67 (1H, s, H-3′), 6.41 (2H, d, *J*** **=** **8.2** **Hz, H-5′,6′), 6.19 (1H, s, H-6), 5.00 (1H, overlapped, H-7″), 4.60 (1H, s, H-4a″), 4.51 (1H, s, H-4b″), 2.88 (2H, m, H-1″), 2.55 (1H, m, H-2″), 2.11 (2H, m, H-6″), 1.69 (3H, s), 1.59 (3H, s), 1.49 (3H, s). ^13^C NMR (125** **MHz, CD_3_OD) *δ* 181.4 (C-4), 162.7, 162.0, 161.4, 159.7, 156.4, 150.4, 149.6, 143.0, 132.4, 130.1, 124.6, 115.2, 111.7, 108.5, 106.8, 106.6, 104.8, 98.4, 32.5 (C-6″), 28.6 (C-2″), 26.0 (C-9″), 19.2 (C-4″), 18.0 (C-10″).

### Compound 4

^1^H-NMR (500** **MHz, CD_3_OD) *δ* 8.06 (1H, d, *J*** **=** **8.2** **Hz, H-5), 7.56 (2H, s, H-2′,6′), 7.42 (2H, d, *J*** **=** **8.2** **Hz, H-3′,5′), 6.86 (1H, m, H-8), 6.82 (1H, d, *J*** **=** **8.2** **Hz, H-6). ^13^C NMR (125** **MHz, CD_3_OD) *δ* 177.7 (C-4), 163.8 (C-7), 160.3 (C-4′), 159.2 (C-8a), 153.8 (C-2), 130.9 (C-2′,6′), 128.3 (C-5), 125.2 (C-3), 124.9 (C-1′), 117.8 (C-4a), 116.1 (C-6), 114.5 (C-3′,5′), 103.0 (C-8), 56.5 (-OMe).

### Compound 5

^1^H-NMR (500** **MHz, CD_3_OD) *δ* 7.31 (2H, d, *J*** **=** **8.2** **Hz, H-2′,6′), 6.81 (2H, d, *J*** **=** **8.2** **Hz, H-3′,5′), 5.91 (1H, s, H-6), 5.39 (1H, dd, *J*** **=** **12.6, 2.9** **Hz, H-2), 5.13 (1H, t, *J*** **=** **7.3** **Hz, H-2″), 3.18 (1H, d, *J*** **=** **7.3** **Hz, H-1″), 3.07 (1H, dd, *J*** **=** **16.8, 12.6** **Hz, H-3a), 2.71 (1H, dd, *J*** **=** **16.8, 2.9** **Hz, H-3b), 1.60 (3H, s, H-5″), 1.56 (3H, s, H-4″). ^13^C NMR (125** **MHz, CD_3_OD) *δ* 198.2 (C-4), 166.4 (C-7), 163.2 (C-8a), 161.7 (C-5), 159.0 (C-4′), 131.6 (C-3″), 131.5 (C-1′), 129.0 (C-2′,6′), 124.1 (C-2″), 116.4 (C-3′,5′), 109.2 (C-8), 103.4 (C-4a), 96.5 (C-6), 80.3 (C-2), 44.1 (C-3), 26.0 (C-5″), 22.6 (C-1″), 18.0 (C-4″).

### Tyrosinase assay

Enzyme assay was performed according to the modified methods in the previous papers^7^. For the calculation of inhibitory activity, 130** **μL of tyrosinase (about 46 units/mL) solvated in 0.1** **mM phosphate buffer (pH: 6.8) and 20** **μL of 1–0.0078** **mM concentrations of the inhibitors were mixed in a 96-well plate, and then 50** **μL of 2** **mM L-tyrosine in buffer was added in mixture. To test the enzyme kinetic study, 130** **μL of tyrosinase and 20** **μL of inhibitor were also mixed, and then 50** **μL of 0.62–10** **mM L-tyrosine was added in a 96-well plate. The mixture was recorded at UV-Vis 475** **nm during 20 min. The inhibitory ratio was calculated according to the following equation:

$$\text{Inhibitory activity rate }\left(\%\right)=100–[\left(S_{\text{2}0}–S_{0}/C_{\text{2}0}–C_{0}\right]\times \text{1}00$$

where *C*_20_ and *S*_20_ are the intensity of control and inhibitor after 20** **min, *C*_0_ and *S*_0_ are the intensity of control and inhibitor at 0 min.

### Molecular docking simulation

This study was performed as previously described with modification^14^. The X-ray structure of tyrosinase was achieved to RCSB (protein data bank ID: 2Y9X) for docking simulation, and then water and substrate molecules in this were deleted. The structure of ligand was constructed by referring 3D structure with Open chemistry database of pubChem in NIH. Non-competitive inhibitors (**1**, **3** and **4**) were performed to form the grid (X: 150, Y: 150, Z: 150) by Autodock 4.2, and competitive inhibitor (**2**) was docked with the grid containing the activity site (number of points: X: 70, Y: 70, Z: 70).

### ABTS radical assay

The ABTS radical cation assay was modified the method of Re et al.^15^ Briefly, the mixture of 7.4** **mM ABTS solution with 2.6** **mM potassium persulfate incubated at room temperature in dark for 24** **h. The ABTS solution diluted with phosphate-buffered saline (pH: 7.4) showed an absorbance of 0.7** **±** **0.03 at 732** **nm. Fifty** ** microliter of the inhibitor was reacted with 950** **μL of the ABTS solution. Absorbance value was monitored during 10** **min at 732** **nm using a spectrophotometer. The free radical value (SC50) was calculated by the log-dose inhibition curve.

Scavenging activity (%)=[1–(extract absorbance/control absorbance)]×100.

### Cell culture

Human hepatoma HepG2 cells were cultured in DMEM supplemented with 10% FBS, 100 units/mL of penicillin and 100** **μg/mL of streptomycin. Cells were maintained incubation at 37** **°C under a 5% CO_2_^16^.

### Measurement of intracellular ROS

The concentration of intracellular ROS was measured using a carboxy-H2DCFDA probe, as previously described^16^. HepG2 cells were seeded into six-well plates at a density of 1** **×** **105 cells/mL and incubated for 24** **h. After treatment with 10** **μM concentrations of inhibitors **1–5** for 1** **h, H_2_O_2_ (2** **mM) was added to plates for 30** **min. Cells were then incubated with 10** **μM carboxy-H2DCFDA for 20** **min, then cells were washed and harvested. Cells were immediately examined using a flow cytometer (Cytomics FC500; Beckman, Miami, FL).

## Results and discussion

### Isolation and identification

**Figure 1.**
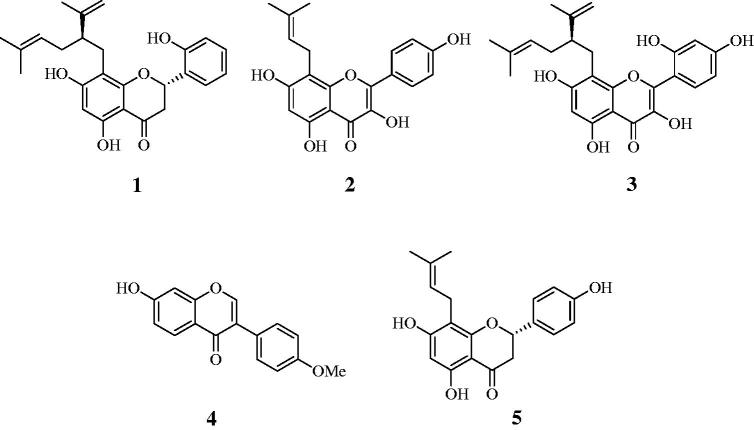
Structures of isolated compounds **1–5** from *S. flavescens*.

A methanol extract of *S. flavescens* roots was progressively partitioned with chloroform, ethyl acetate and water fractions. Ethyl acetate was subjected to silica gel and C-18 column chromatography using organic solvents to obtain five compounds (**1–5**). Their structures were identified by comparing the spectroscopic results with those previously published, and the compounds were determined to be kushenol A (**1**)^17^, 8-prenylkaempferol (**2**)^18^, kushenol C (**3**)^18^, formononetin (**4**)^5^ and 8-prenylnaringenin (**5**) (Figure 1)^19^.

### Enzyme assay

**Figure 2.**
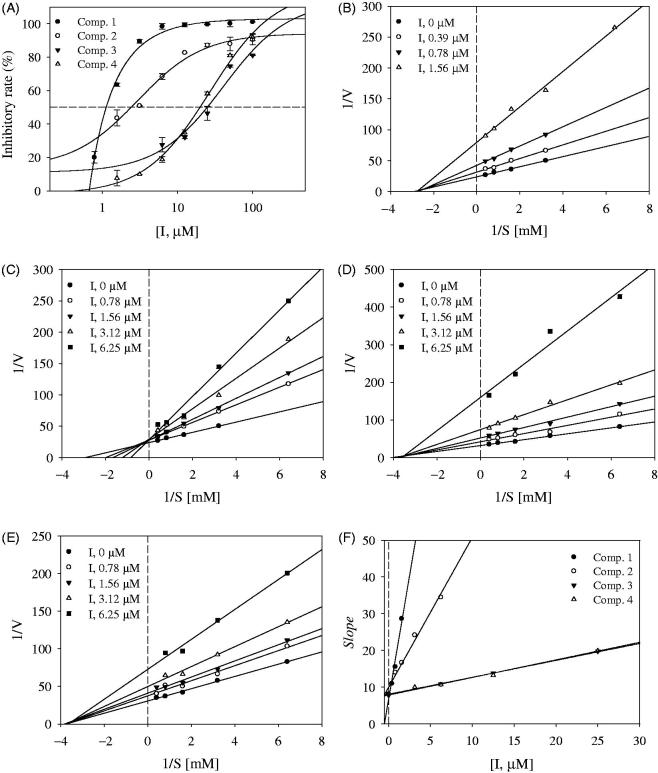
(A) Inhibitory activity of compounds **1–4** on tyrosinase. (B–E) Linewever-Burk plots of tyrosinase inhibition by compounds **1–4**, respectively. (F) Secondary re-plot of slope vs. [I].

All of the compounds (**1–5**) were tested for inhibitory activity, as evidenced by a decrease in the amount of l-dihydroxyphenylalanine produced from l-tyrosine by tyrosinase using an ultraviolet-visible (UV-Vis) photometer at 475** **nm. Kojic acid was used as a positive control (IC50 value: 16.7** **±** **2.4** **μM). Among them, compounds **1–4**, which exhibited over a 80% inhibitory rate at 100** **μM, were tested *in vitro* at a range of concentrations using a UV-Vis spectrophotometer to determine their IC50 values. As shown in Figure 2(A) and Table 1, compounds **1–4** displayed tyrosinase inhibitory activity in a dose-dependent manner, with IC50 values ranging from 1.1** **±** **0.7 μM to 24.1** **±** **2.3** **μM. According to these results, compounds **1** and **2** may act as potential inhibitors of tyrosinase, with IC50 values of 1.1** **±** **0.7 μM and 2.4** **±** **1.1** **μM, respectively. Previous report revealed that chalcone derivatives, kuraridinol and kuraridin, showed high inhibitory activity, with IC50 values of 0.8 and 0.6 μM^12^^,^^13^. Also, main components of prenylated flavonoids, sophoraflavanone G and kurarinone, possessed the inhibitory activity, with IC50 values of 6.6 and 6.2** **μM, respectively^12^^,^^13^. Although compounds **1** and **2** showed lower inhibitory activity on the catalytic reaction of tyrosinase than those of chalcone derivatives, they have worth having insight as a tyrosinase inhibitor because they regulate enzymes within a few micromole.

### Enzyme kinetics

This study confirmed the inhibitory mechanism of compounds **1–4** towards tyrosinase using enzyme kinetics. Enzyme kinetics were performed based on academic methods that examine a variety of substrate concentrations. The results were represented by Lineweaver–Burk plots, which confirmed the inhibition to involve a two-step binding mechanism. The Lineweaver–Burk plots of inhibitors **1**, **3** and **4** showed a series of straight lines passing through a point on the negative abscissa (non-competitive inhibitor). In contrast, all of the straight lines produced by different concentrations of inhibitor **2** passed through a point on the axis of ordinates (competitive inhibitor). Finally, the Ki values calculated for inhibitors **1–4** were 0.4** **±** **0.4 μM, 2.4** **±** **0.1 μM, 16.0** **±** **0.3 μM, and 17.1** **±** **1.1** **μM, respectively, on the secondary re-plot (Figure 2(F) and Table 1).

**Table 1.** Tyrosinase inhibitory activities of compounds **1–5** and their enzyme kinetics.

	Inhibitory activity of compounds on tyrosinase^a^		
	100 μM (%)	IC50 (μM)	Binding mode (*K_i_*, μM)
**1**	101.1 ± 3.3	1.1 ± 0.7	Non-competitive (0.4 ± 0.4)
**2**	90.4 ± 3.0	2.4 ± 1.1	Competitive (2.4 ± 0.1)
**3**	81.1 ± 0.1	24.1 ± 2.3	Non-competitive (16.0 ± 0.3)
**4**	91.9 ± 2.0	19.9 ± 1.7	Non-competitive (17.1 ± 1.1)
**5**	27.0 ± 2.9	N.T.	N.T.
Kojic acid^b^	75.5 ± 3.7	16.7 ± 2.4	

^a^All compounds examined in a set of triplicated experiment.

^b^Positive control.

### Molecular docking simulation

**Figure 3.**
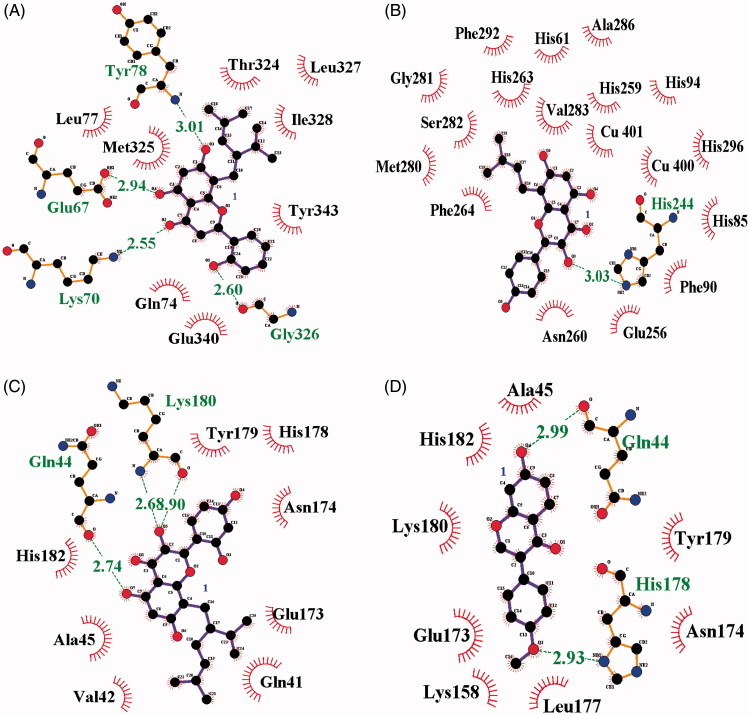
(A–D) The green dotted line present hydrogen bond interactions between ligands **1–4** and receptor, respectively.

**Figure 4.**
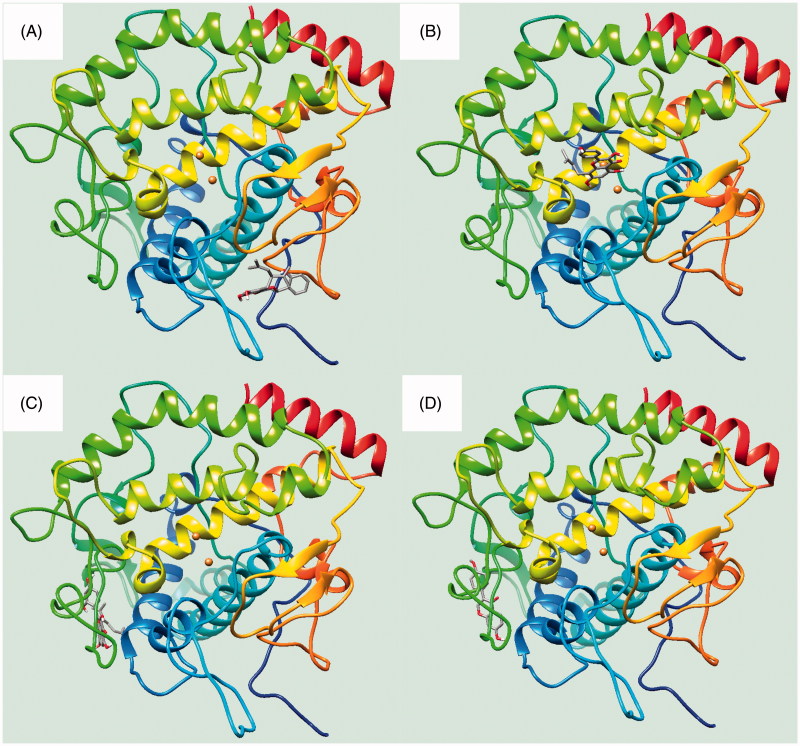
(A–D) The location of respective ligands **1–4** bound into receptor.

To visualise the binding of the receptor to the ligand, inhibitors were subjected to molecular docking analysis set up with their respective grids and included both the tyrosinase (**1**, **3** and **4**) and active site (**2**) based on the enzyme kinetics data. As indicated in Figure 3 and Table 2, inhibitors **1–4** were docked onto tyrosinase via hydrogen bonds, with a stable binding score calculated by Autodock 4.2. Among them, compound **1** had the lowest binding energy of –7.13** **kcal/mol, with hydrogen bonds among residues Gln67 (2.94** **Å), Lys70 (2.55** **Å), Tyr78 (3.01** **Å) and Gly326 (2.60** **Å) in left coil site of tyrosinase (Figure 4(A)). However, compound **2**, which showed the second lowest inhibitory activity of 2.4** **±** **1.1** **μM *in vitro*, had the highest binding energy (–6.77** **kcal/mol). When comparing compound **2** with the other inhibitors, it is important to remember that this is a competitive inhibitor in active site (Figure 4(B)). Inhibitor **2** was docked to the active site via hydrogen bonds to His244 (3.03** **Å).

**Table 2.** Interaction and Autodock score between tyrosinase and inhibitors.

	Hydrogen bonds (Å)	Binding energy (kcal/mol)
**1**	Gln67 (2.94), Lys70 (2.55), Tyr78 (3.01), Gly326 (2.60)	–7.13
**2**	His244 (3.03)	–6.77
**3**	Gln44 (2.74), Lys180 (2.68, 2.90)	–6.86
**4**	Gln44 (2.99), His178 (2.93)	–7.07

Additionally, compound **3**, with each binding energies of –6.86, took three hydrogen bonding with Gln44 (2.74** **Å) and Lys180 (2.68 and 2.90** **Å) into right coil site of tyrosinase. Also, compound **4** had the interaction to Gln44 (2.99** **Å) and His178 (2.93** **Å) at –7.07** **kcal/mol Autodock score (Figure 4(D)). Still now, known potential inhibitors have been suggested predicted binding pose into active site through in silico^7^^,^^9^^,^^20^^,^^21^. These have given the hint to find new competitive inhibitor for developing skin whiting^7^^,^^9^^,^^20^^,^^21^. Molecular docking study unveiled the possible position as two allosteric sites of tyrosinase by prenylated flavonoid. Moreover, ligand bonded into left coil site maybe give more effect to the catalytic reaction than that of right coil site. Figure 4(A–D) shows the location where inhibitors **1–4** are bound to the receptor.

### ABTS radical-scavenging activity

The antioxidant activity of all of the isolated compounds **1–5** was evaluated using the ABTS radical-scavenging assay. The compounds exhibited over 50% scavenging activity at a concentration of 25** **μM. As shown in Table 3, compounds **1–3** and **5** exhibited highly potent inhibitory activity on ABTS+** **radical scavenging, with IC50 values of 9.7** **±** **0.1 μM, 7.9** **±** **0.3 μM, 4.9** **±** **0.3 μM, and 7.0** **±** **0.2** **μM, respectively.

**Table 3.** Scavenging activity of compounds on ABTS radical.

	Scavenging activity of compounds on ABTS radical^a^	
	25 μM (%)	SC50 (μM)
**1**	93.7 ± 0.3	9.7 ± 0.1
**2**	93.2 ± 0.2	7.9 ± 0.3
**3**	94.1 ± 0.2	4.9 ± 0.3
**4**	53.3 ± 2.4	22.8 ± 1.1
**5**	91.5 ± 0.1	7.0 ± 0.2
Ascorbic acid^b^	44.9 ± 0.9	27.8 ± 0.5

^a^All compounds examined in a set of triplicated experiment.

^b^Positive control.

### Measurement of intracellular ROS

**Figure 5.**
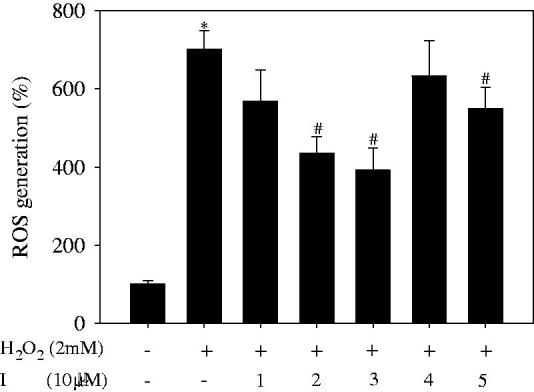
Effect of compounds **1–5** on intracellular ROS generation in H_2_O_2_-treated H2P2G cells (The results are presented as the means** **± SDs of three replicates of on represent experiment. *p** **< .05 vs. negative group, #*p* < .05 vs. positive group).

To examine their antioxidant activity *in vitro*, pure compounds **1–5** were added to HepG2 cells, followed by addition of 2** **mM H_2_O_2_. As shown in Figure 5(A), the respective negative (H_2_O_2_: –; I: –) and positive (H_2_O_2_:**  **+; I: –) controls generated reactive oxygen species (ROS) of 100.0%** **±** **9.1% and 701.5%** **±** **46.9%. Among the tested compounds, compounds **2** and **3** caused a decrease in ROS generation of 435.3%** **±** **43.0% and 391.7%** **±** **56.3%, respectively, compared with the negative control. The viability of HepG2 cells following treatment with all compounds **1–5** at 10** **μM ranged from 100.1%** **±** **6.1% to 103.8%** **±** **5.1% (Figure S6). Compound **2** caused an approximately 10% increase in cell viability relative to the positive control.

## Conclusion

*S. flavescens* has been used not only as a medicine and functional food for hundreds of years, but also as a pesticide to manage various insects^2^^,^^22^. The main components of this plant were recently determined to exert inhibitory activity on tyrosinase^12^^,^^13^. Based on this information, this study aimed to identify compounds from *S. flavescens* that block catalysis reaction of this enzyme. Five compounds – kushenol A (**1**), 8-prenylkaempferol (**2**), kushenol C (**3**), formononetin (**4**) and 8-prenylnaringenin (**5**) – were isolated by column chromatography (silica gel and C-18 resins) from the ethyl acetate fraction of the methanol extract of *S. flavescens* roots. Among the isolated compounds that displayed strong inhibitory activity on tyrosinase *in vitro* (**1–4**), compounds **1** and **2** showed the highest inhibitory activity, with IC50 values of 1.1** **±** **0.7 and 2.4** **±** **1.1, respectively. Furthermore, the combined research of enzyme kinetics and molecular docking was performed to identify the binding positions of four of the compounds (**1–4**). With the exception of a competitive inhibitor preferring selection combining into the active site, compounds **1**, **3** and **4** were determined to be non-competitive inhibitors bound to the allosteric site. Compound **1** displayed more inhibitory activity on tyrosinase than did compounds **3** and **4**. Additionally, in silico, compound **1** was bound to the left coil site, whereas compounds **3** and **4** were bound to the right coil site; these results were confirmed by the Autodock scores, which corresponded to their IC50 values. These facts suggest detail study to be necessary on left coil site for developing tyrosinase inhibitor of non-competitive type. There is a high possibility that this may be an allosteric site on moiety of compound **2**. However, compound **2** had a lower Autodock score than those of the non-competitive inhibitors. One reason for this result may be that compound **2** operates to block enzyme catalysis in direct competition with the substrate. Through the research of ROS scavenging in relation to Skin aging^23^, compounds **1**–**3** and **5** exhibited highly potent scavenging activity on the ABT radical cation; moreover, compounds **2** and **3** diminished the level of ROS produced by HepG2 cells treated with H_2_O_2_. Finally, kushenol A (**1**) and 8-prenylkaempferol (**2**) were confirmed as potential inhibitors of enzymes targeted by cosmetics for skin whitening and aging, and by insect control.

## Supplementary Material

Supplemental Material
