# Supplementary material for: Kushenol A and 8-prenylkaempferol, tyrosinase inhibitors, derived from Sophora flavescens
Source: J Enzyme Inhib Med Chem. 2018 Jun 6;33(1):1048–54. doi: 10.1080/14756366.2018.1477776 (PMC6009905; doi:10.1080/14756366.2018.1477776)
Supplement: Supplemental Material [file IENZ_A_1477776_SM3891.pdf]

Kushenol A and 8-prenylkaempferol, tyrosinase inhibitors, derived from *Sophora flavescens*

Jang Hoon Kim[a,c], In Sook Cho[b], Yang Kang So[a], Hyeong-Hwan Kim[b,]**, Young Ho Kim[c,]**

[a]*Advanced Radiation Technology Institute, Korea Atomic Energy Research Insititute, Jeongeup, Jeollabuk 56212, Republic of Korea*

[b]*Department of Horticultural and Crop Environment, National Institute of Horticultural and Herbal Science, RDA, Wanju, 55365, Republic of Korea*

[c]*College of Pharmacy, Chungnam National University, Daejeon 34134, Republic of Korea*

**Co-Corresponding authors

Tel.: +82 42 821 5933 (Y.H. Kim); fax: +82 42 823 6566 (Y.H. Kim); e-mail addresses: yhk@cnu.ac.kr (Y.H. Kim).

Tel.: +82-63-238-6333 (Kim, H. H.); fax: +82-63-238~~-6305~~ (Kim, H. H.); e-mail addresses: hhkim8753@korea.kr

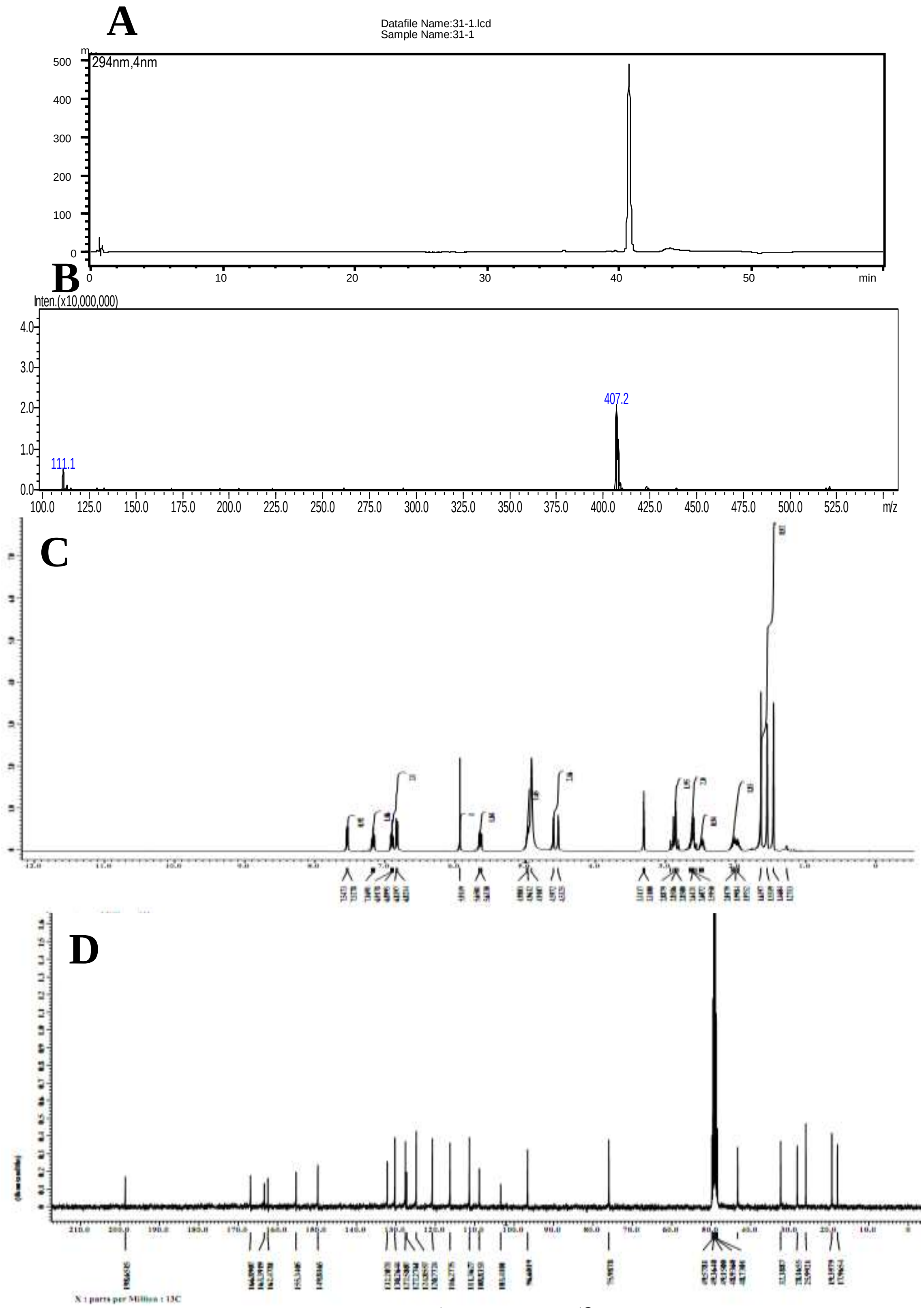

Figure S1. HPLC (A), HR-ESI-MS (B), ^{1}H (C)-, and ^{13}C (D)-NMR spectra of compound **1**. **(**CD_3OD-*d*$_4$ at 500 MHz)

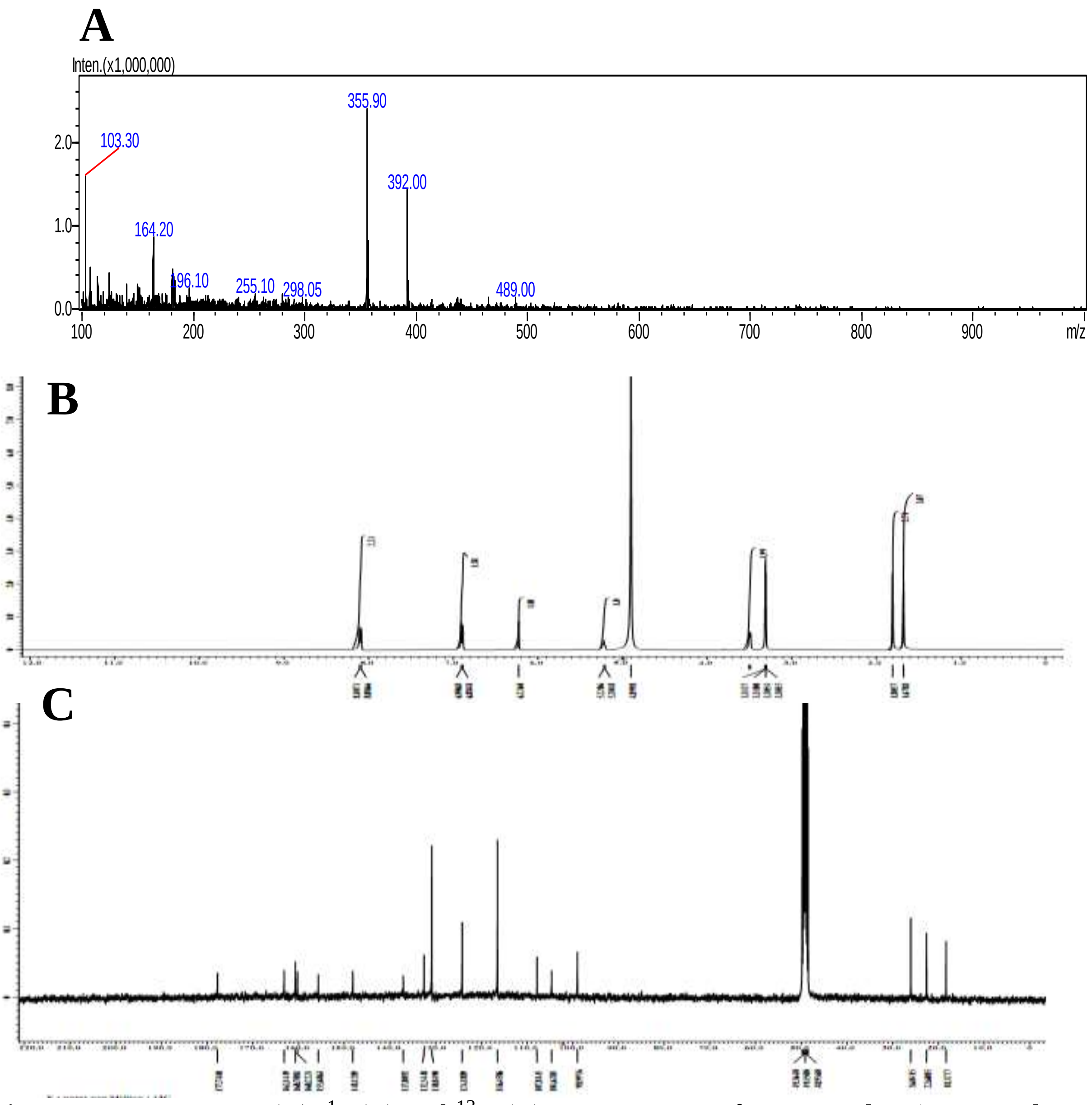

Figure S2. HR-ESI-MS(A), ^{1}H(B)-and ^{13}C (C)-NMR spectra of compound **2**. **(**CD_3OD-d_4 at 500 MHz)

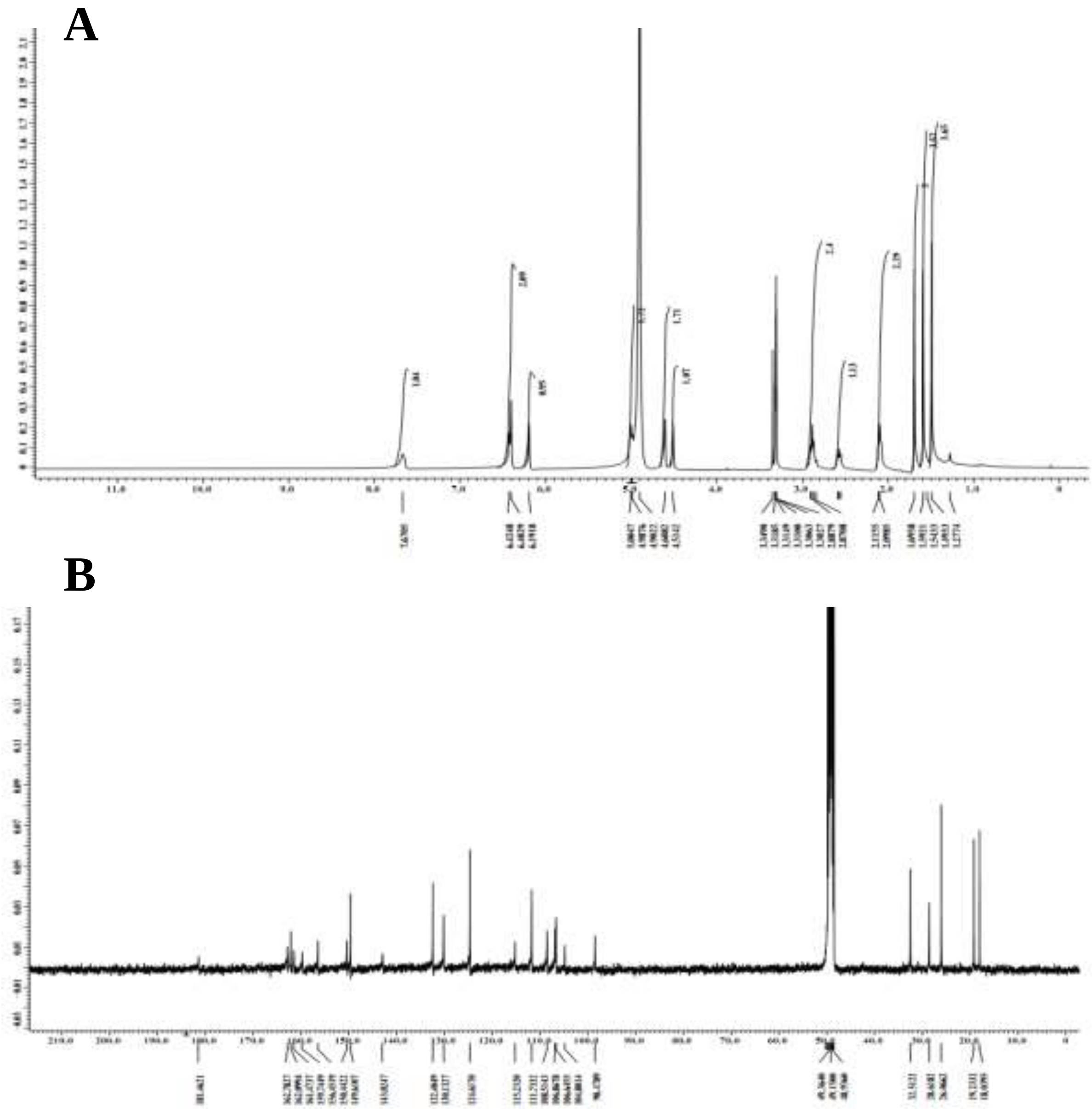

Figure S3. ^{1}H (A)-and ^{13}C (B)-NMR spectra of compound **3**. **(**CD_3OD-d_4 at 500 MHz)

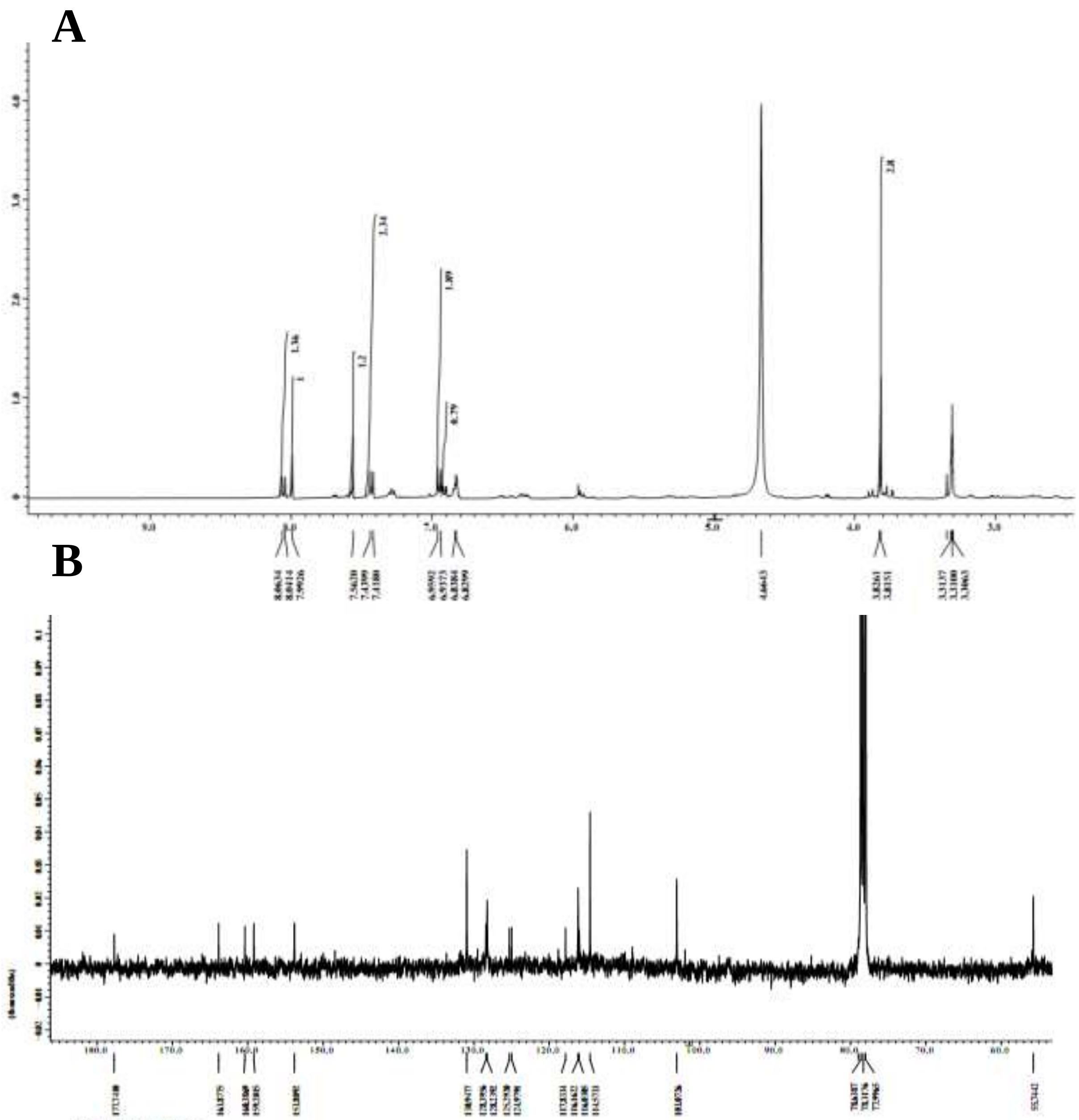

Figure S4. ^{1}H (A)-and ^{13}C (B)-NMR spectra of compound **4**. **(**CD_3OD-d_4 at 500 MHz)

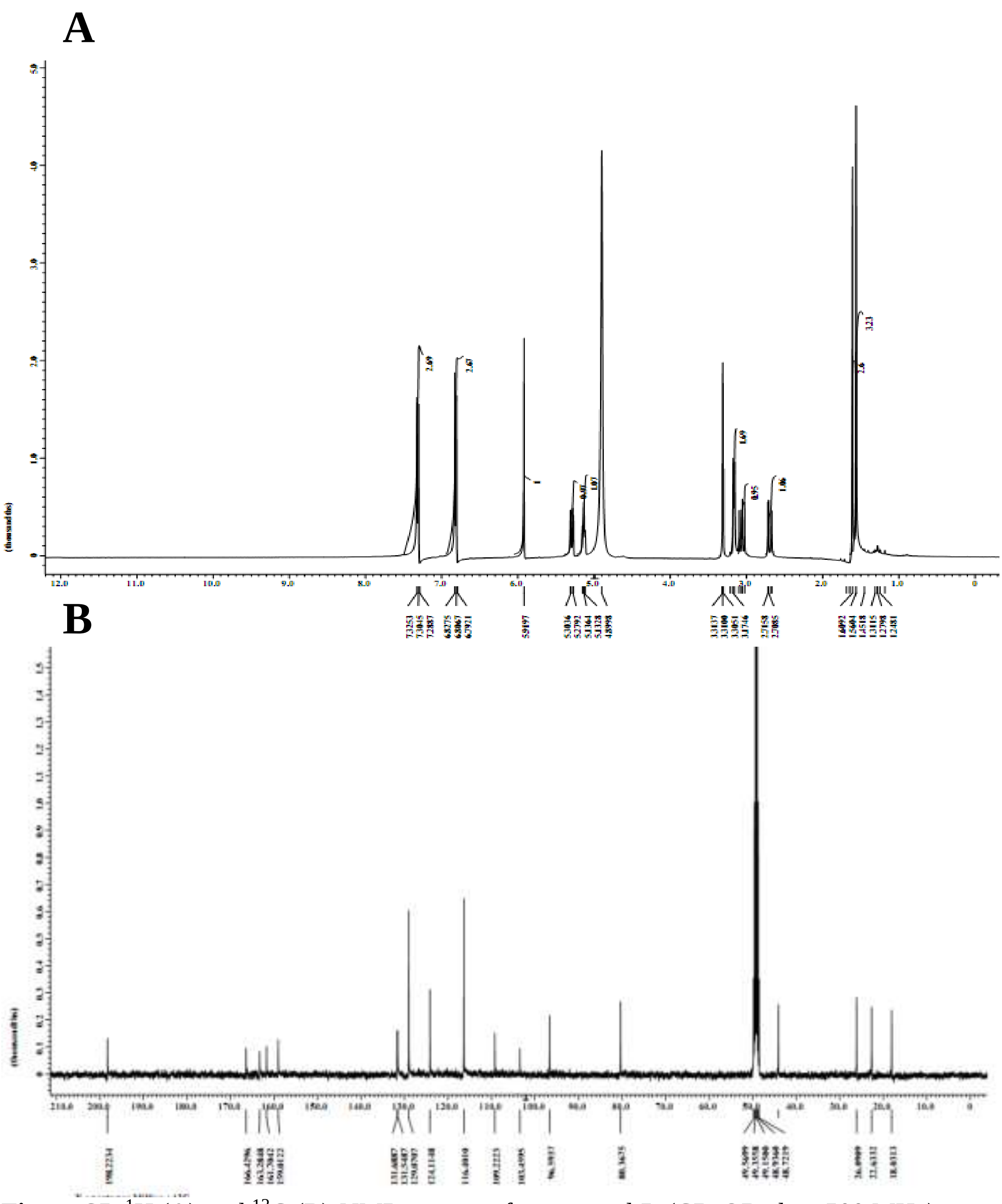

Figure S5. ^{1}H (A)-and ^{13}C (B)-NMR spectra of compound **5**. **(**CD_3OD-d_4 at 500 MHz)

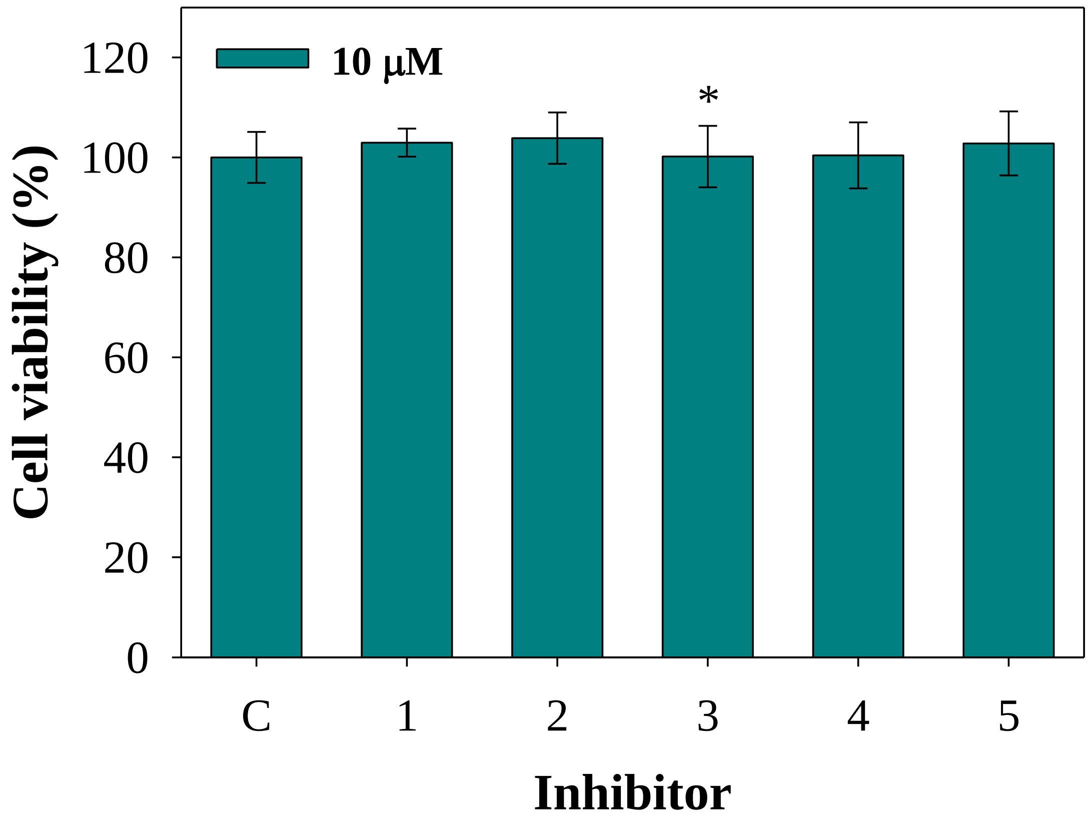

Figure S6. Cytotoxicity of compounds **1-5** at 10 μM against Human hepatoma HepG2 cells. (The results are presented as the means ± SDs of six replicates of on represent experiment. *p<0.05 vs. positive group.)

Cell viability was measured using an EZCytox cell viability assay kit (Daeil Lab, Seoul, Korea) according to the manufacturer's protocol. Briefly, cells were cultured in a 96-well plate at a density of 1×105 cells/mL for 24 h. Cells were subsequently treated with 10 μM concentrations of inhibitors (**1-5**). Cells were incubated for an additional 24 h, after which 10 μL of the kit solution was added to each well, followed by incubation for 4 h at 37oC under a 5% CO2. The index of cell viability was determined based on measurement of formazan production using an ELISA reader (Benchmark Plus; Bio-Rad) at an absorbance of 480 nm (Cho et al., 2015)

References

Cho B-O, Ryu H-W, Lee C-W, Jin C-H, Seo W-D, Ryn JH, Kim D-S, Kang SY, Yook H-S, Jeong I-Y.. Protective effects of new blackberry cultivar MNU-32 extracts against H_2O_2-induced oxidative stress in HepG2 cells. Food Sci Biotech 2015;24:643-50.
